# Viral Metagenome-Based Precision Surveillance of Pig Population at Large Scale Reveals Viromic Signatures of Sample Types and Influence of Farming Management on Pig Virome

**DOI:** 10.1128/mSystems.00420-21

**Published:** 2021-06-08

**Authors:** Biao He, Wenjie Gong, Xiaomin Yan, Zihan Zhao, Ling’en Yang, Zhizhou Tan, Lin Xu, Aiwei Zhu, Jianing Zhang, Jihong Rao, Xinglong Yu, Jianfeng Jiang, Zongji Lu, Yifang Zhang, Jianmin Wu, Yong Li, Yuxiang Shi, Qing Jiang, Xiwen Chen, Changchun Tu

**Affiliations:** a Key Laboratory of Jilin Province for Zoonosis Prevention and Control, Institute of Military Veterinary Medicine, Academy of Military Medical Sciences, Changchun, Jilin Province, China; b College of Veterinary Medicine, Hunan Agricultural University, Changsha, Hunan Province, China; c College of Life Sciences and Engineering, Foshan University, Foshan, Guangdong Province, China; d College of Veterinary Medicine, Yunnan Agricultural University, Kunming, Yunnan Province, China; e Guangxi Key Laboratory of Veterinary Biotechnology, Guangxi Veterinary Research Institute, Nanning, Guangxi Zhuang Autonomous Region, China; f College of Life Sciences, Ningxia University, Yinchuan, Ningxia Hui Autonomous Region, China; g College of Life Sciences and Food Engineering, Hebei University of Engineering, Handan, Hebei Province, China; h Zoonoses Institute, College of Veterinary Medicine, Jilin University, Changchun, Jilin Province, China; i Center of Animal Disease Prevention and Control of Rongchang District, Rongchang, Chongqing Municipality, China; j Animal Disease Prevention and Control & Healthy Breeding Engineering Technology Research Center, Mianyang Normal University, Mianyang, Sichuan Province, China; k Jiangsu Co-Innovation Center for Prevention and Control of Important Animal Infectious Diseases and Zoonosis, Yangzhou University, Yangzhou, Jiangsu Province, China; Johns Hopkins Bloomberg School of Public Health

**Keywords:** pigs, biosafety, farm management, organ specificity, precision surveillance, virome

## Abstract

Pigs are a major meat source worldwide and a pillar of Chinese animal husbandry; hence, their health and safety are a prioritized concern of the national economy. Although pig viruses have been continuously investigated, the full extent of the pig virome has remained unknown and emerging viruses are still a major threat to the pig industry. Here, we report a comprehensive study to delineate the pig virome of 1,841 healthy weaned pigs from 45 commercial farms collected from 25 major pig-producing regions across China. A viromic sequence data set, named Pigs_VIRES, which matched 96,586 viral genes from at least 249 genera within 66 families and which almost tripled the number of previously published pig viromic genes, was established. The majority of the mammalian viruses were closely related to currently known ones. A comparison with previously published viromes of bovines, avians, and humans has revealed the distinct composition of Pigs_VIRES, which has provided characteristic viromic signatures of serum, pharyngeal, and anal samples that were significantly influenced by farming management and disease control measures. Taken together, Pigs_VIRES has revealed the most complete viromic data set of healthy pigs to date. The compiled data also provide useful guidance to pig viral disease control and prevention and the biosafety management of pig farms. Especially, the established viromic protocol has created a precision surveillance strategy to potentially innovate currently used surveillance methods of animal infectious diseases, particularly by making precision surveillance available to other animal species on a large scale or even during a nationwide surveillance campaign.

**IMPORTANCE** Pigs are deeply involved in human lives; hence, their viruses are associated with public health. Here, we established the most comprehensive virome of healthy piglets to date, which provides a viromic baseline of weaned pigs for disease prevention and control, highlighting that longitudinal viromic monitoring is needed to better understand the dynamics of the virome in pig development and disease occurrence. The present study also shows how high standards of animal farm management with strict biosafety measures can significantly minimize the risk of introduction of pathogenic viruses into pig farms. Particularly, the viromic strategy established, i.e., high-throughput detection and analyses of various known and unknown pathogenic viruses in a single test at large scale, has completely innovated current surveillance measures in provision of timely and precise detection of all potentially existing pathogenic viruses and can be widely applied in other animal species.

## INTRODUCTION

Domesticated pigs are a major meat source for humans worldwide and thus provide concerns for global food safety. China has the largest pig farming industry in the world, with more than 700 million annual slaughters since 2013, accounting for >55% of the world total (1, 2). China also imports tens of thousands of breeder pigs every year, mainly from the United States, Canada, and some European countries (3), making the Chinese pig population very heterogeneous. However, pig farming on such a large scale and the frequent introduction of foreign pig breeds pose a major challenge for the control and prevention of pig diseases. With the rapid development of economy and increasing demand for pork, Chinese pig farming is undergoing transformation and upgrading from the traditional free-range mode to intensified industrialized production under popularization of the healthy feeding concept. However, viral diseases are still the main threat to the pig industry. Mutated or new viruses continue to emerge and have caused outbreaks severely affecting the pig industry, such as variant porcine epidemic diarrhea virus (PEDV) and porcine pseudorabies virus (PRV), porcine deltacoronavirus, swine acute diarrhea syndrome-coronavirus (SADS-CoV), and porcine circovirus 3 (PCV3) (4–8). Moreover, exotic diseases have also caused devastating consequences to pig farmers, such as the incursion and subsequent spread of African swine fever in China (9). In addition, pigs are recognized as important hosts that transmit zoonotic viruses to humans (10), such as hepatitis E, Nipah, influenza A, and Japanese encephalitis viruses (11–14). To address the food safety and public health concerns associated with pig diseases and to minimize the transmission of pig-associated zoonotic viruses to humans, traditional technology in disease surveillance using pathogen-specific tests does not meet the demand for large-scale and high-throughput screening of various pathogens in a single or several tests, especially since the pathogen-specific tests are unable to discover the highly mutated, new, or exotic ones. To meet the demand, a comprehensive study to understand the viromic background of pig viruses, especially the mammalian ones, and their genetic diversity was necessary.

Pig viruses have been investigated for about a hundred years, using traditional isolation or PCR methods (15). In recent years, some progress to elucidate the pig virome has been made by using high-throughput sequencing (HTS)-based viral metagenomic analyses (16–18). However, such investigations have been limited in scope and mainly focused on finding the possible causative agents of certain diseases. The full extent of the global pig virome has remained unknown. Here, we report a comprehensive virome profile of more than a thousand healthy piglets from 45 commercial pig farms across China, and the results reveal the most complete viromic structure of a health animal population to date. In addition, the resulting viromic data set and technical protocol can be used to establish precision surveillance strategy, which will innovate the traditional surveillance methods of pig viral diseases on a large scale and serve as an example for surveillance of other animal diseases as well.

## RESULTS

### Establishment and quality assessment of the pig viromic data set.

**FIG 1:**
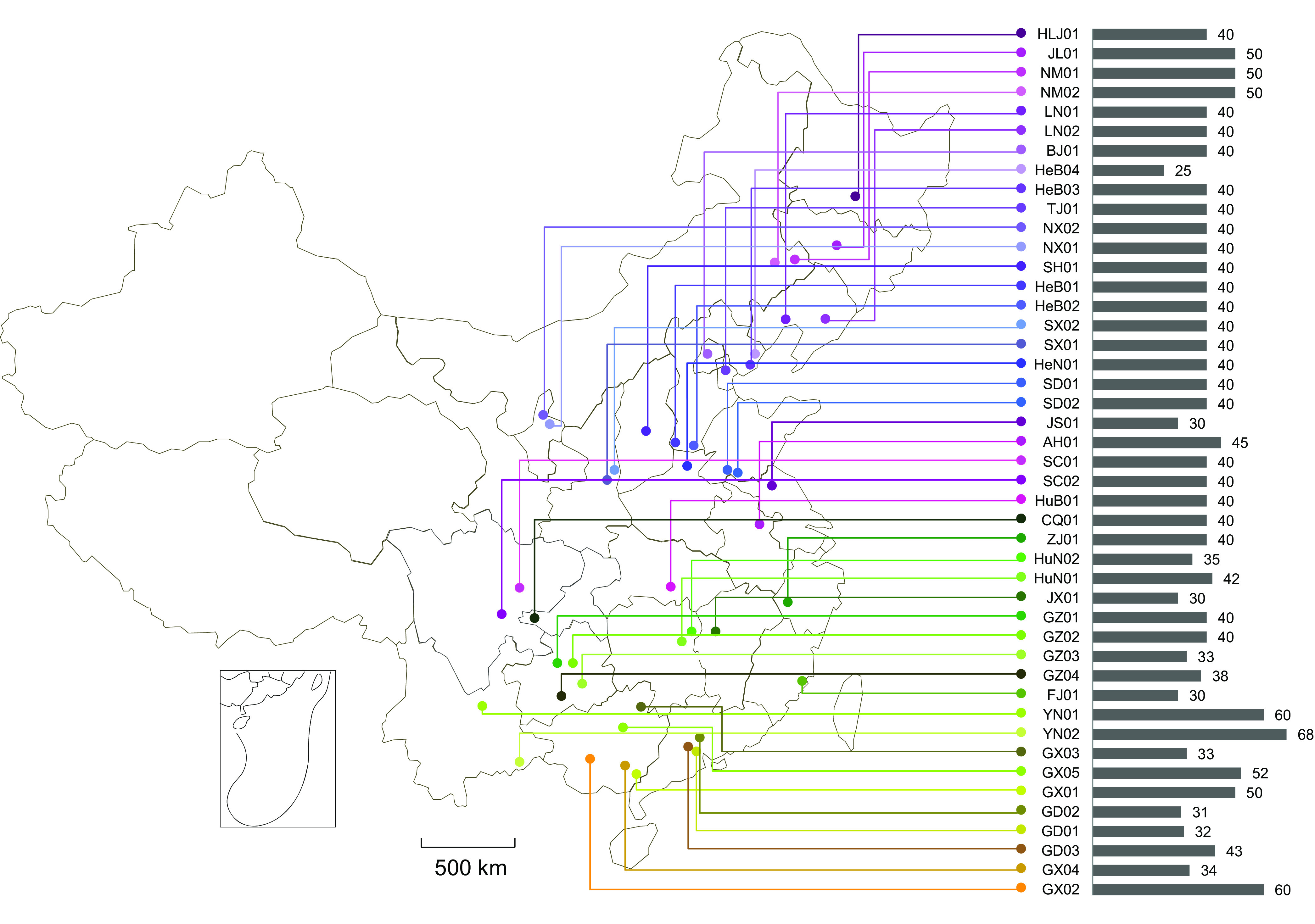
Geographical distribution of 45 sampled pig farms. Horizontal bars indicate the numbers of pigs sampled in each farm.

From 1 April to 20 May 2017, we collected 5,523 pharyngeal and anal swabs and serum samples from 1,841 healthy weaned piglets (35 to 50 days old) from 45 farms in 25 major pig-producing regions of China (Fig. 1). The viral nucleic acids (NA) prepared from these samples were subjected to HTS, yielding 100.9 gigabases (Gb) of high-quality data (average, 2.2 Gb per farm) (see Data Set S1 in the supplemental material). For viral metagenomic analyses (Fig. S1), host gene sequences (∼1.0%) (Data Set S1) were removed and the remaining reads were sequentially subjected to *de novo* assembly to produce contigs, followed by reference-based and hidden Markov model (HMM)-based virus annotation. This process generated the preliminary viromic data set (PVD), containing 4,543,977 virus-like sequences (VLSs) (including contigs and reads). After BLASTn/x validation against the nt/nr database, 3,791,933 VLSs were retained in the PVD, with the majority of the removed VLSs being bacteriophage-like sequences (BLSs) (*n* = 533,856) that were highly similar to bacterial genomes, followed by phycodnavirus- and retrovirus-like sequences showing significant similarities to mammalian genomes.

10.1128/mSystems.00420-21.1 FIG S1 Schematic pipeline of sample preprocessing (A) and viromic bioinformatic annotation (B). *, in this study, we used a 0.22-μm-pore-size membrane to remove host cell debris and bacteria. To investigate large viruses, a membrane of 0.45 μm pore size or larger is recommended. **, here we constructed an overlapping PE125 library, but another library with a different insert size can be used. ***, our inserts here were overlapped by sequencing reads; hence, reads can be merged according to overlapping region using FLASH. If inserts were longer than 2 times the sequencing length, this step should be omitted. Download FIG S1, EPS file, 2.2 MB. Copyright © 2021 He et al. 2021 He et al. https://creativecommons.org/licenses/by/4.0/ This content is distributed under the terms of the Creative Commons Attribution 4.0 International license.

10.1128/mSystems.00420-21.5 DATA SET S1 Summary of Illumina sequencing and virus annotation. Download Data Set S1, XLSX file, 0.01 MB. Copyright © 2021 He et al. 2021 He et al. https://creativecommons.org/licenses/by/4.0/ This content is distributed under the terms of the Creative Commons Attribution 4.0 International license.

Since contamination with foreign VLSs might be introduced into HTS-based viromic analyses due to sample preparation using certain laboratory components (19–22), viral false-positive sequences (FPSs) derived from nucleic acid extraction spin columns and laboratory components were retrieved from GenBank (20, 22) and used to further check the potential contamination in the PVD, which identified a small number of FPSs related to simian virus 40 (SV40) in the data sets from farms GZ03 (*n* = 7), GZ04 (*n* = 17), NX01 (*n* = 9), NX02 (*n* = 7), and SX02 (*n* = 149), indicating the potential presence of laboratory component-derived (LCD) contamination. Therefore, these sequences were removed. The same search of our control samples (bat, rodent, and tick samples processed with the same protocols at the same time in our laboratory) and other published viromes (Data Set S2) also revealed widely distributed reads related to SV40, human adenovirus C (HAdV-C), and parvo-like hybrid virus (PHV), e.g., SV40 in Canadian cattle (CAN_Cattle_2018; *n* = 11) and Chinese pigs (CHN_Pigs_2018; *n* = 675), HAdV-C in U.S. broiler chickens (USA-HUN_Broilers_2016; *n* = 20) and CHN_Pigs_2018 (*n* = 1,116), and PHV in Swedish piglets (SWE_Pigs_2016; *n* = 25), indicating a necessary removal of LCD sequence from HTS data. To check intersample cross-contamination, comparison with viromes of the above-described control samples did not find any pig virus-specific sequences, and no sequences of these viromes were found in the PVD, indicating no cross-contamination. However, a very small number of viral sequences (*n* = 559) related to several bacteriophages in the PVD were found in all three viromes. These were therefore considered to be FPSs and were also removed.

10.1128/mSystems.00420-21.6 DATA SET S2 Details of data set used in this study to assess the Pigs_VIRES virome. Download Data Set S2, XLSX file, 0.01 MB. Copyright © 2021 He et al. 2021 He et al. https://creativecommons.org/licenses/by/4.0/ This content is distributed under the terms of the Creative Commons Attribution 4.0 International license.

Furthermore, some human endogenous retrovirus (HERV) H-like reads were also identified in 95.6% (43/45) of the farms, with 80.0 to 100% nucleotide (nt) similarities. To confirm if they were true or contaminated sequences, PCR of the HERV *gag-pol* gene showed 49.4% ± 8.3% (mean ± standard deviation [SD]) HERV DNA-positive results in serum samples from four selected farms (JL01, NM01, SC02, and SD01). PCR analysis of the pig genome extracted from ear tissue of a Duroc pig did not produce positive amplification of the HERV gene fragment, indicating that the HERV genome was not integrated into the pig genome. To determine whether these HERV-like sequences (HLSs) originated from a human retrovirus transmitted to pig or from LCD contamination, we conducted a further search of HLSs against our nonpig viromic data sets, as well as against other data sets from China and other countries (Data Set S2). Results showed that HLSs were extensively present in all these data sets, with 80 to 100% nucleotide similarities: e.g., 390 reads in FM1701, 14,941 in XB1703, 37 in SWE_Pigs_2016, 217 in USA_Human_2015, and 6,798 in CAN_Cattle_2019, etc. These results indicate that the HLSs were more likely to be foreign FPSs than human-transmitted HERV and were therefore excluded. These results emphasize that quality assessment is essential in an HTS-based virome, requiring that the sequence data sets must be carefully scrutinized to remove any potential viral FPS contamination. After removal of FPSs through the above-described analyses, the remaining VLSs in PVD were condensed at a nucleotide similarity of 95%, and the eventual pig virome was generated and named Pigs_VIRES.

**FIG 2:**
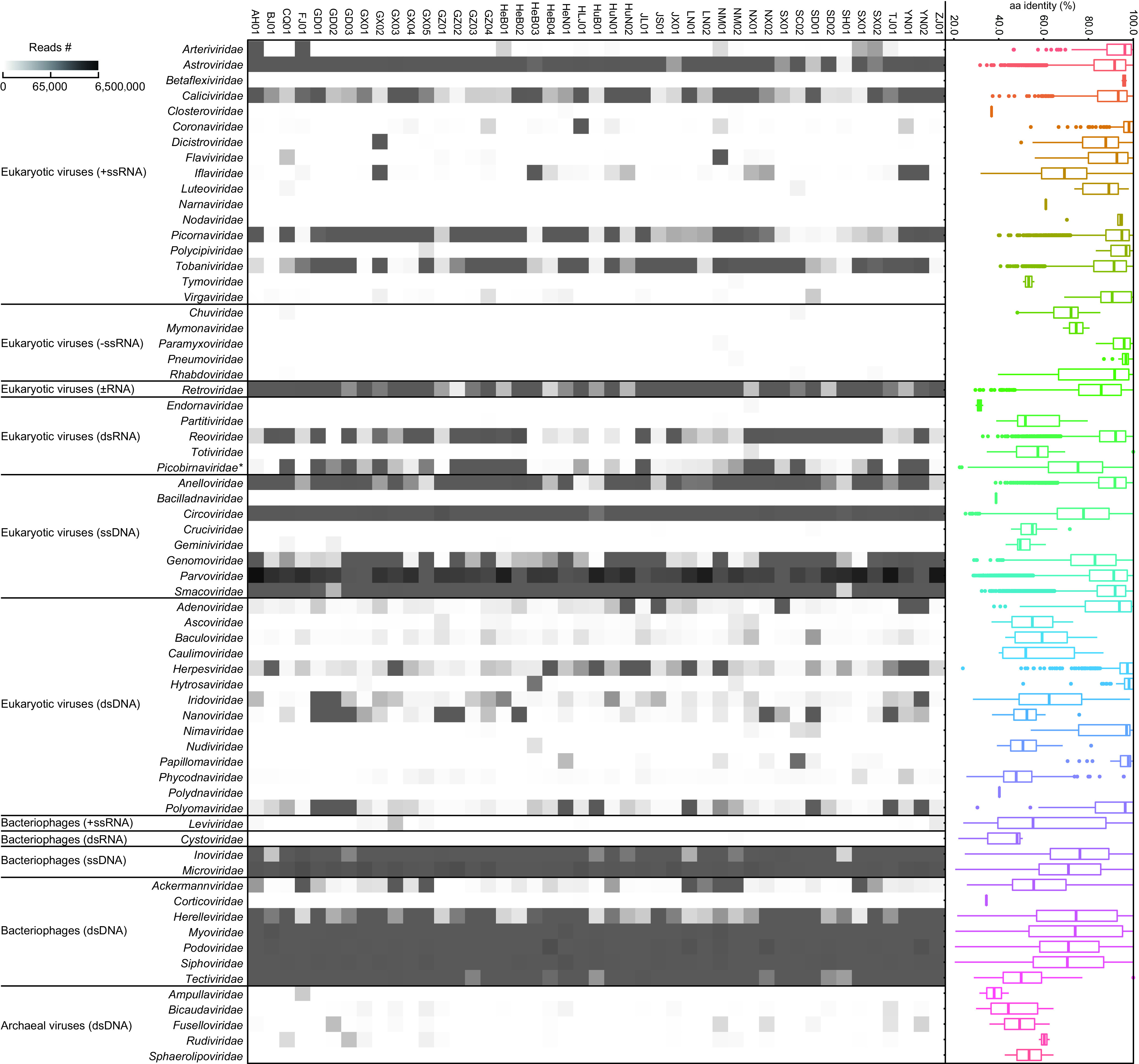
Overview of Pig_VIRES. Left panel, heat map of viral reads from 45 pig farms (horizontal) catalogued into 11 groups of viral families (vertical) according to viral hosts and genome types (+ssRNA, single-stranded positive RNA; −ssRNA, single-stranded negative RNA; dsRNA, double-stranded RNA; ±ssRNA, reverse-transcribed single-stranded RNA; ssDNA, single-stranded DNA; dsDNA, double-stranded DNA). Right panel, amino acid identity range of the reads in the given viral family with reference sequences in GenBank. Each box plot illustrates the estimated median (center line), upper and lower quartiles (box limits), and outliers (points) of the similarity. Picobirnaviridae* indicates that this was classified as a eukaryotic viral family in this study, although extensively conserved prokaryotic ribosomal binding sites are found in picobirnaviruses (72).

Overall, Pigs_VIRES contained 244,246 assembled contigs (including 271 circular viral genomes) and 1,099,169 unassembled reads. Among these contigs, the majority (90.6%, *n* = 221,383) were obtained from nucleotide/amino acid reference-based annotation, while only 9.4% (*n* = 22,863) came from remote HMM-based prediction. Pigs_VIRES represents at least 96,586 viral genes in GenBank within at least 30 RNA and 36 DNA viral families known to infect eukaryotic, bacterial, and archaeal organisms (Fig. 2). The number of viral reads per family per farm is summarized in Data Set S3. Of these VLSs, 52.8% shared 87.6% ± 11.4% amino acid similarities with known eukaryotic viruses, with the remaining 47.2% sharing 71.3 ± 17.4% amino acid similarities with prokaryotic viruses. Further taxonomic assignment classified Pigs_VIRES into 109 eukaryotic and 140 prokaryotic viral genera (43.8 ± 6.9 and 51.0 ± 11.8 per farm, respectively) (Data Set S4). Among the eukaryotic viral genera, 30 (27.5%) were highly prevalent and present in more than 80% of farms and 12 (11.0%) were moderately prevalent and present in 40% to 80% of farms, with the remaining 67 (61.5%) less prevalent and present in less than 40% of farms. Compared to 3.5% (5,666,371) of RNA viral reads in total (161,181,496), DNA viral reads were overwhelmingly abundant, accounting for 96.5% (Data Set S3). The highest numbers of viral reads were obtained within the families *Parvoviridae* (2,583,787 ± 1,711,191 reads per farm), *Microviridae* (344,625 ± 287,803), *Podoviridae* (143,619 ± 201,560), and *Siphoviridae* (116,686 ± 97,601). Viruses of the families *Myoviridae*, *Circoviridae*, *Reoviridae*, *Inoviridae*, *Tobaniviridae*, *Astroviridae*, and *Picornaviridae*, etc., also had tens of thousands of reads per farm (Data Set S3). In general, our viral metagenomic protocol generated high proportions of virus-like reads, with a range of 3.7% to 40.4% of total reads (20.5% ± 8.5% per farm) (Data Set S1).

10.1128/mSystems.00420-21.7 DATA SET S3 Overview of reads numbers per viral family per farm. Download Data Set S3, XLSX file, 0.02 MB. Copyright © 2021 He et al. 2021 He et al. https://creativecommons.org/licenses/by/4.0/ This content is distributed under the terms of the Creative Commons Attribution 4.0 International license.

10.1128/mSystems.00420-21.8 DATA SET S4 Overview of reads number of genus level cluster per body sample. Download Data Set S4, XLSX file, 0.1 MB. Copyright © 2021 He et al. 2021 He et al. https://creativecommons.org/licenses/by/4.0/ This content is distributed under the terms of the Creative Commons Attribution 4.0 International license.

### Pigs_VIRES significantly expanded the existing pig viromic genes.

**FIG 3:**
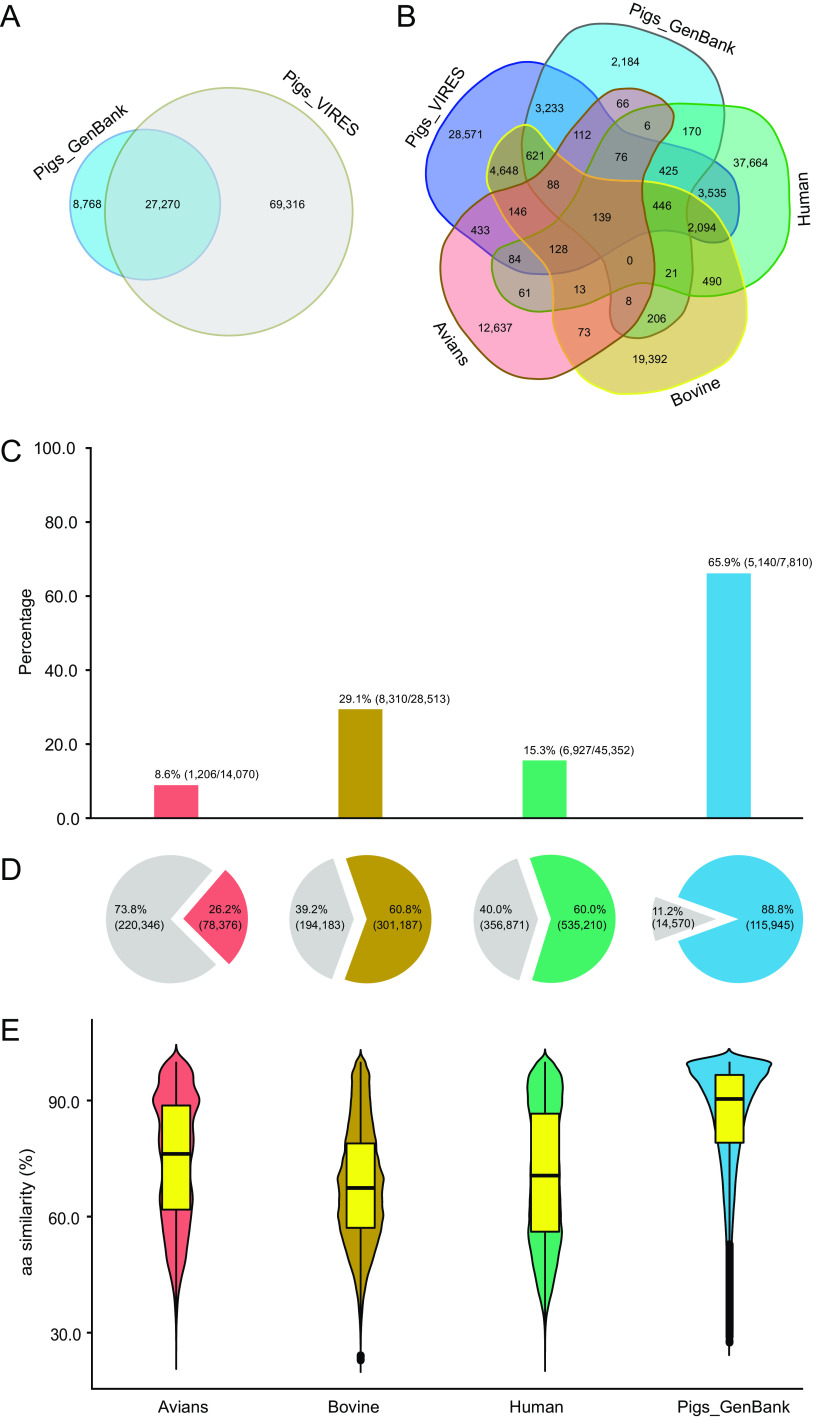
Comparison of Pigs_VIRES with published human and other animal viromes. (A) Specific and shared viral gene numbers of Pigs_VIRES and Pigs_GenBank. (B) Specific and shared protein cluster (PC) numbers among Pigs_VIRES and other viromes. (C) Percentage of Pigs_VIRES-sharing PC numbers of the other viromes relative to their total PC numbers. (D) Percentage of other viromic VLS numbers unmatched (gray) and matched (non-gray colors) to Pigs_VIRES. (E) Amino acid (aa) similarities of matched VLSs between Pigs_VIRES and other viromes. The identifiers of each item in panels C to E are shown at the bottom of the figure.

We compared Pigs_VIRES with published viromes (Data Set S2) of pigs, bovines (takins, cattle, and goats), avians, and humans by using the pipeline of the present study. Results showed that 4 previously published pig viromes generated, in total, 130,515 VLSs and annotated to 36,038 viral genes in GenBank (referred to as Pigs_GenBank). As shown in Fig. 3A, 75.7% of genes (27,270/36,038) in Pigs_GenBank overlapped those in Pigs_VIRES, with 84.3% being prokaryotic and the remainder mainly classified as eukaryotic picornavirus, astrovirus, parvovirus, and circovirus, etc. Although 71.8% of the genes (69,316/96,586) in Pigs_VIRES were not reported in the published pig viromes with the prokaryotic genes being predominant (83.7%) and the remaining being eukaryotic, mainly rotavirus, arterivirus, adenovirus, and circovirus, etc., due to the large scale of sampling, Pigs_VIRES has almost triple the number of previously known pig viromic genes. Finally, the two data sets were combined into one larger pig viromic data set, referred to as Pigs_Virome, which can be used as the most complete pig viromic sequence assemblage up to date.

To compare Pigs_VIRES with other published viromes of human and other animals, the predicted protein sequences of all those viromes were pooled and clustered using the Markov cluster tool (MCL) within vConTACT2. Results showed that 63.8% (28,571/44,779) of Pigs_VIRES protein clusters (PCs) were specific to Pigs_VIRES (Fig. 3B), with 65.9%, 29.1%, 15.3%, and 8.6% of PCs in Pigs_GenBank, bovine, human, and avian viromes, respectively, shared with Pigs_VIRES (Fig. 3C). These Pigs_VIRES-specific PCs comprised eukaryotic rotaviruses, toroviruses, teschoviruses, enteroviruses, porcine parvoviruses, and circoviruses, etc., and also included prokaryotic microviruses, inoviruses, myoviruses, and podoviruses, etc., while the shared PCs between Pigs_VIRES and bovine, human, and avian viromes were mainly prokaryotic microviruses (38.5%) and caudoviruses (26.2%) and eukaryotic parvoviruses (14.7%) and unclassified circoviruses (4.0%). Further sequence comparison showed that 88.8%, 60.0%, 60.8%, and 26.2% of VLSs in Pigs_GenBank, human, bovine, and avian viromes, respectively, matched those in Pigs_VIRES (Fig. 3D) and shared 86.0% ± 13.8%, 70.7% ± 17.5%, 68.3% ± 14.9%, and 74.6% ± 16.2% amino acid similarities, respectively (Fig. 3E). The Pigs_VIRES sequences divergent (<50% amino acid similarity) from Pigs_GenBank were predominantly (82.2%) prokaryotic caudoviruses and microviruses, with the minority being the eukaryotic viruses, such as picornaviruses, genomoviruses, astroviruses, sapoviruses, and anelloviruses, while sequences with ≥80% amino acid similarity to human, bovine, and avian viromes, besides caudoviruses and microviruses, included parvoviruses, rotaviruses, and smacoviruses, etc. These results showed that Pigs_VIRES covered the majority of VLSs in the other pig viromes and that different animal species had distinct viromic compositions.

### Genetic diversity of mammalian viruses in the pig virome.

Many mammalian viruses, such as classical swine fever virus (CSFV), porcine reproductive and respiratory syndrome virus (PRRSV), PCV2, and PEDV, pose a major threat to the pig industry. Vaccinations against these pathogenic viruses are widely applied in China (Data Set S5), and therefore all mammalian VLSs were searched using BLASTn against the genomes of live vaccine strains to differentiate the vaccine sequences. Results revealed that besides field strains, some vaccine sequences of PRRSV, PEDV, CSFV, and PRV, but not transmissible gastroenteritis virus (TGEV), were present (Data Set S6). It is interesting to note that both field viruses and vaccine strains cocirculated in some farms, such as PEDV in farm HLJ01 and PRRSV in farm AH01 (Data Set S6), which should be especially concerning, since it is possible that new viruses could be generated through potential recombination between field viruses and vaccine strains, which would result in vaccine failures. To further understand the genetic diversity of mammalian viruses, the 2,214 representatives of mammalian viral contigs in Pigs_VIRES were classified into 39 groups within 17 viral families (Fig. S2). In all, 82.4% of the contigs shared ≥85.0% nucleotide similarity with known pig viruses, and the remaining 17.6% contigs had <85.0% similarity to astrovirus, sapovirus, torovirus, pasivirus, teschovirus, enterovirus, picobirnavirus, rotavirus, anellovirus, circular DNA virus, and polyomavirus and probably represented new viruses (Fig. S2). Maximum-likelihood phylogenies also showed that pathogenic viruses such as CSFV, PRRSV, PEDV, and TGEV and the most abundant parvoviruses did not show significant variation and were closely related to current field strains. Here, PCV1 and PCV3 were not found in these samples, but reads of pathogenic PCV2 were prevalent in ∼50% of the farms (22/45) and shared >98.0% similarity with current strains. It is interesting to note that some sequences from farm NX01 showed the highest 73% nucleotide similarity with a mink circovirus (MiCV) (23), indicative of a new circovirus of pigs. Further PCR screening showed that 12.5% of anal and 10.0% of pharyngeal samples, but no serum samples, were positive for the same amplicons. Phylogenetic analysis showed that these sequences were grouped together with the most recently reported novel porcine circovirus 4 (PCV4) detected in Hunan Province, China (24), and hence were the same virus species.

10.1128/mSystems.00420-21.2 FIG S2 The assembled 2,214 mammalian virus contigs were assigned to 39 groups showing ≥85.0% (gray) or lower (orange) nucleotide similarities with currently known viruses. Download FIG S2, EPS file, 1.6 MB. Copyright © 2021 He et al. 2021 He et al. https://creativecommons.org/licenses/by/4.0/ This content is distributed under the terms of the Creative Commons Attribution 4.0 International license.

10.1128/mSystems.00420-21.9 DATA SET S5 Sample details, immunization records, and scoring of management and biosafety measures of farms. Download Data Set S5, XLSX file, 0.01 MB. Copyright © 2021 He et al. 2021 He et al. https://creativecommons.org/licenses/by/4.0/ This content is distributed under the terms of the Creative Commons Attribution 4.0 International license.

10.1128/mSystems.00420-21.10 DATA SET S6 BLASTn searches against complete vaccine genomes using the Pigs_VIRES VLSs of each farm. Download Data Set S6, XLSX file, 0.03 MB. Copyright © 2021 He et al. 2021 He et al. https://creativecommons.org/licenses/by/4.0/ This content is distributed under the terms of the Creative Commons Attribution 4.0 International license.

### Genetic diversity and host prediction of bacteriophages.

**FIG 4:**
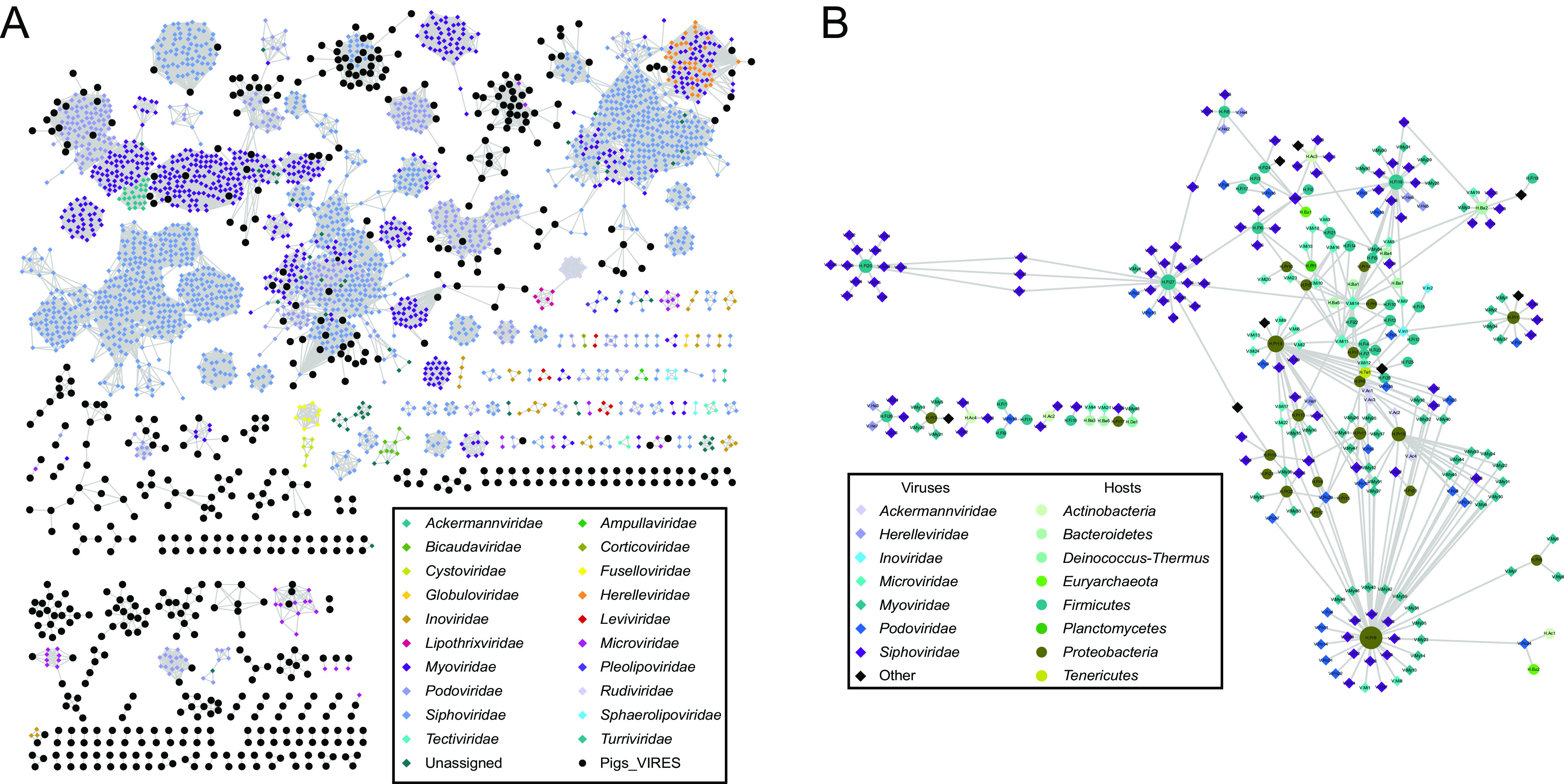
Genus-level clustering network of BLSs (black circles) with prokaryotic RefSeq references (colored diamonds) (A) and predicted bacterial hosts (colored circles) of BLSs (color-filled diamonds) (B).

Bacteriophages constituted a large part of Pigs_VIRES and represented the most highly divergent and abundant viruses (Fig. 2). The BLSs separated into 140 viral clusters (VCs) at the approximate genus level and 242,437 singletons. Of these VCs, only 15 (10.7%) clustered within known families such as *Myoviridae*, *Podoviridae*, *Siphoviridae*, and *Microviridae*, while the majority (89.3%) were novel (Fig. 4A and Data Set S3). We used the MVP database (25) and CRISPR spacers to predict hosts for bacteriophages, allowing us to identify putative hosts for 6,895 sequences mainly from *Myoviridae* (41.1%), *Podoviridae* (25.4%), and *Siphoviridae* (16.1%). About 76.8% BLSs matched one host, with 18.1% and 5.1% matching two and three, respectively. These putative hosts included at least 137 species and 63 genera within 42 families, 24 orders, 17 classes, and 8 phyla. The majority of hosts were *Firmicutes* (*n* = 59) and *Proteobacteria* (*n* = 48), followed by *Bacteroidetes* (*n* = 17), *Actinobacteria* (*n* = 8), *Euryarchaeota* (*n* = 2), *Deinococcus-Thermus* (*n* = 1), *Planctomycetes* (*n* = 1), and *Tenericutes* (*n* = 1) (Fig. 4B).

### Viromic signatures of the body samples.

**FIG 5:**
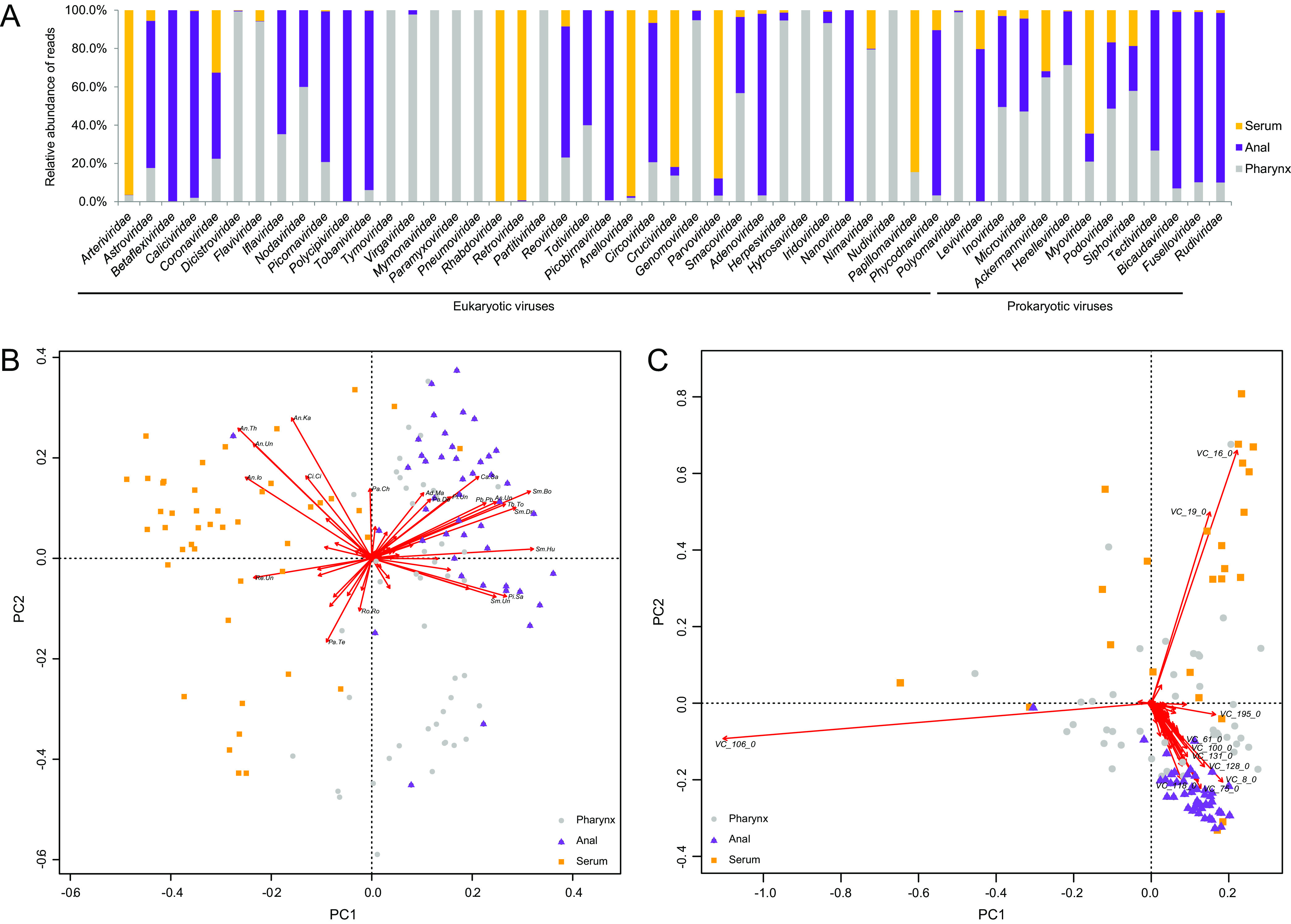
Viromic signatures of the body samples. (A) Relative VLS abundance in serum, anal, and pharyngeal samples at the viral family level; (B and C) PCA analyses of Jaccard distances of eukaryotic (B) and prokaryotic (C) viromic binary composition at the level of genus clusters in pharyngeal (gray circles), anal (purple triangles), and serum (orange squares) samples. Annotated genera are indicated by directional arrows, and names are shown if the absolute values of PC1 and/or PC2 scores of a genus were >0.1. The abbreviations of the genus cluster names are given in Data Set S4 in the supplemental material.

At the family level, viral reads varied in abundance in different body samples: e.g., reads in *Arteriviridae*, *Retroviridae*, and *Papillomaviridae* were mainly in serum samples, reads in *Astroviridae*, *Tobaniviridae*, and *Adenoviridae* were mainly in anal samples, and reads in *Paramyxoviridae*, *Pneumoviridae*, and *Polyomaviridae* were mainly from the pharynx, indicative of body sample specificity of viromic composition (Fig. 5A). Principal-component analysis (PCA) of the eukaryotic virome confirmed this for serum samples and anal swabs, showing high interfarm similarities, but dispersion was noted among pharyngeal samples (Fig. 5B). Some viruses were highly body sample specific: e.g., picobirnaviruses, toroviruses, mastadenovirus, and sapoviruses displayed a tropism for anal swabs (Fig. 5B), while retroviruses, iotatorqueviruses, circoviruses, and tetraparvoviruses were found preferentially in serum samples. In contrast, viral genus tropisms for pharyngeal swabs were not obvious. The PCA of the prokaryotic virome showed a much closer clustering of anal samples with more specific VCs than of pharyngeal and serum samples (Fig. 5C). These results delineated the viromic signatures of the body samples or organ systems.

### Impacts of geographic locations, farm management, and biosafety on the virome.

To investigate the impact of geographic location on the pig virome, eukaryotic and prokaryotic viromic similarities (VS) in all farms were tested by Ward hierarchical clustering, which clustered the 45 pig farms into five VS groups (Ce/p1 to Ce/p5) (Fig. S3). The eukaryotic and prokaryotic clustering did not show correlation with their geographic locations. For instance, farms GD01 and LN01 were 2,200 km apart but showed similar viromic compositions and clustered together in Ce3 and Cp2, respectively, while farms SX01 and SX02 were just 60 km apart but clustered in Ce1 and Ce4 and in Cp5 and Cp3, respectively, showing distinct viromic compositions. This was further noted by redundancy analysis of the principal coordinates of neighborhood matrix (PCNM) of spatial distances and detrended annotation (analysis of variance [ANOVA] test, *P* > 0.05), which also showed that the viromic composition of each farm was not influenced by its geographic location.

10.1128/mSystems.00420-21.3 FIG S3 Ward hierarchical clustering (upper panel) and PCA analysis (lower panel) of Jaccard distance of the general eukaryotic (A) and prokaryotic (B) viromic binary composition at the level of genus cluster showing that the pig virome composition was not influenced geographically. Download FIG S3, PDF file, 0.2 MB. Copyright © 2021 He et al. 2021 He et al. https://creativecommons.org/licenses/by/4.0/ This content is distributed under the terms of the Creative Commons Attribution 4.0 International license.

**FIG 6:**
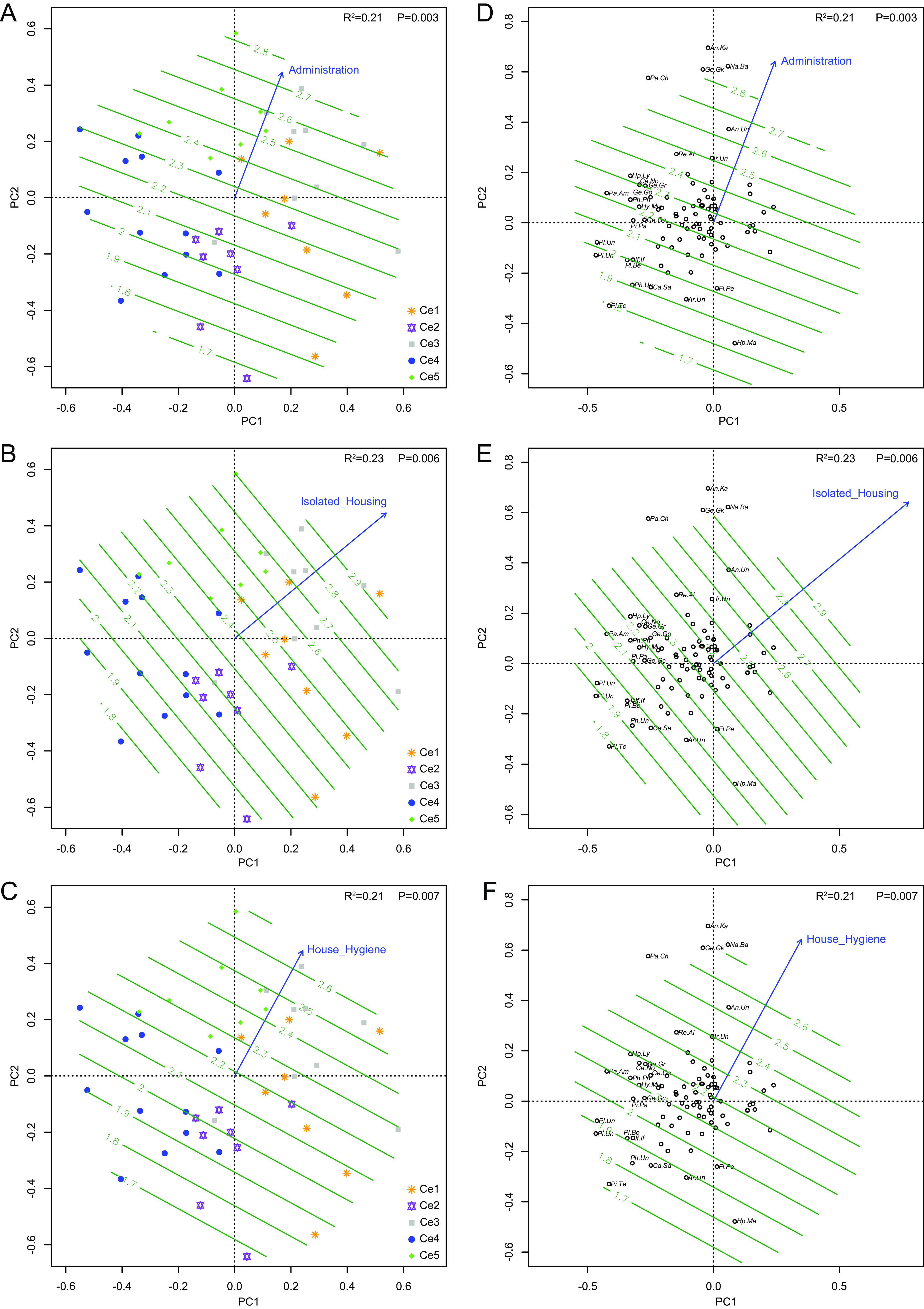
Farms of different groups clustered using Ward hierarchical clustering analysis of the eukaryotic virome showing different scores of the environmental factors of administration (A), isolated housing (B), and house hygiene (C) that exerted highly significant impacts (*P* < 0.01) on the eukaryotic virome (D to F). Viral genera of representatives are listed if the absolute values of PC1 and/or PC2 scores of a genus were >0.2. Impacts of immunization, disinfection, air quality, and quarantine (*P* < 0.05) on the eukaryotic virome are not shown. Genus cluster name abbreviations are given in Data Set S4 in the supplemental material.

**FIG 7:**
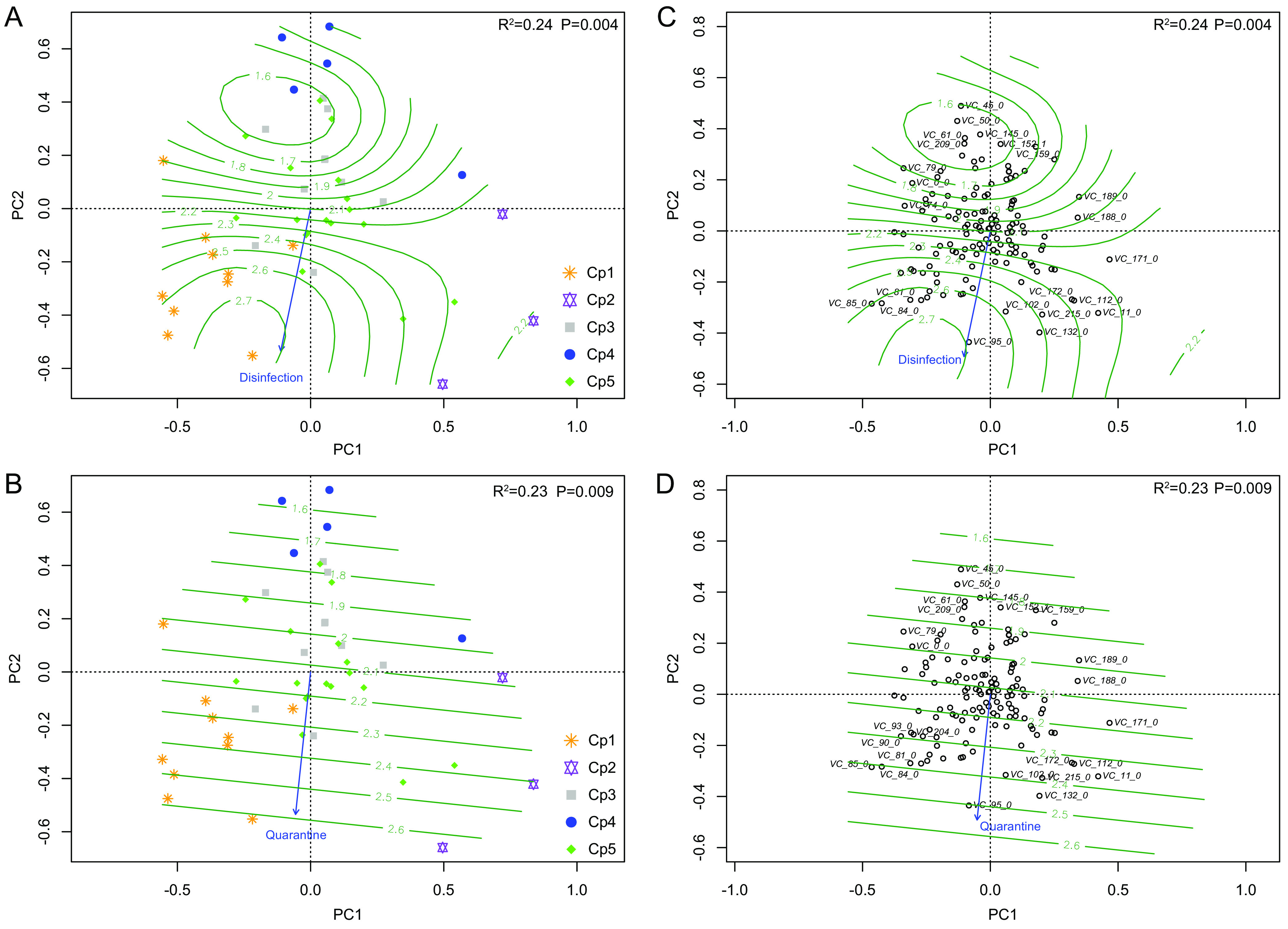
Farms of different groups clustered using Ward hierarchical clustering analysis of the prokaryotic virome showing different scores of the environmental factors of disinfection (A) and quarantine (B) that exerted highly significant impacts (*P* < 0.01) on the prokaryotic virome (C and D). Viral genera of representatives are listed if the absolute values of PC1 and/or PC2 scores of a genus were >0.2. Impacts of air quality, administration, and isolated housing (*P* < 0.05) are not shown. Genus cluster name abbreviations are given in Data Set S4 in the supplemental material.

To evaluate the impact of farming measures on viromic composition, a modified BioCheck protocol (available at www.biocheck.ugent.be) was used to investigate the levels of farm management and biosafety (Table S1 and Data Set S5). Results showed that farms in Ce5 and Ce3 had higher scores in administration, isolated housing, disinfection, immunization levels, hygiene, quarantine, and air quality for the eukaryotic virome and in Cp1 for the prokaryotic virome, while lower scores were found in farms in Ce2 and Ce4 for the eukaryotic virome and in Cp4 for the prokaryotic virome (Fig. 6A to C and 7A and B). Among these covariates, administration, isolated housing, and hygiene had extremely significant impacts (*P* < 0.01) on virome composition (Fig. 6D to F), with immunization, disinfection, air quality, and quarantine all having significant impacts (*P* < 0.05). However, the surrounding environment of the farms had less impact (*P* > 0.05). Babuviruses, anelloviruses, and chapparvoviruses tended to be present in farms with a higher score of environment factors (Fig. 6D to F), while pestiviruses, arteriviruses, teschoviruses, and sapoviruses were more prevalent in farms with lower scores (Fig. 6D to F). However, five covariates, disinfection, quarantine, air quality, administration, and isolated housing, all impacted the prokaryotic virome, with the first two showing extremely significant impacts (Fig. 7).

10.1128/mSystems.00420-21.4 TABLE S1 Questionnaire of management and biosafety measures. Download Table S1, PDF file, 0.06 MB. Copyright © 2021 He et al. 2021 He et al. https://creativecommons.org/licenses/by/4.0/ This content is distributed under the terms of the Creative Commons Attribution 4.0 International license.

## DISCUSSION

Here, we aimed to construct the complete viromic profile of pigs. To maximize virus discovery, serum, anal, and pharyngeal samples from 1,841 healthy weaned piglets representing 45 farms in 25 major pig-producing regions were collected (Fig. 1), since the bloodstream, anus, and pharynx are major sites for viral infection or shedding. The reason we focused on weaned piglets is because they comprise a very important population, not only being the most vulnerable to virus infections and environmental change but also being sources to produce sows and fattening pigs. Moreover, viromes of neonatal and adult pigs have previously been reported but their scales were small (16–18, 26). Results showed that Pigs_VIRES covered the majority of, and significantly expanded previous lists of, pig viromic genes and provides the most complete viromic background of the healthy pig population (Fig. 2), which can help identify and monitor emerging viruses. Given that commercial pig farming is the main stream of contemporary animal husbandry in the world and that China is the largest player, with its pigs having diverse import sources (2, 3, 27), the viromic data obtained here may largely represent the global swine virome. Of note is that the ∼94 viral genera captured in each farm was much less than the total 249 genera in Pigs_VIRES, suggesting that the viromic data generated by pooled samples of ∼40 pigs per farm may not well represent the virus diversity of each farm; hence, a large scale of investigation is necessary to obtain the viromic panorama of pigs.

The present study showed that 82.4% of mammalian viruses are highly similar to known pig viruses (see Fig. S2 in the supplemental material), probably due to previously intensive identification and investigation of these viruses. Most of these viruses, with limited or unknown pathogenicity, were prevalent in over 80% of farms, including non-PPV1 parvoviruses, non-PCV circular DNA viruses, anelloviruses, sapeloviruses, toroviruses, mamastroviruses, picobirnaviruses, non-group A rotaviruses, and enteroviruses, and hence can be considered resident viruses. An emerging concept is that the virome may contain mutualistic symbiotes (28–32): e.g., chronic gammaherpesvirus 68 infection in mice might benefit the host to resist pathogenic challenges (33), indicating that such non- or less-pathogenic viruses may provide unexpected beneficial effects to the host (34). Like probiotics that help hosts against pathogenic bacteria, resident viruses may play roles in maintaining the health status of hosts. In contrast, there were nonresident viruses that were not as prevalent and abundant, with many, such as CSFV, PCV2, and PRRSV, being able to infect pigs and cause diseases and therefore being pathogenic.

Bacteriophages constitute a large part of Pigs_VIRES and also dominate in the human virome (35). Intensive pig husbandry is believed to be a major source of antibiotic resistance in bacteria (36, 37), and phages have become a renewed approach to combat antibiotic-resistant bacteria (38). In Pigs_VIRES, these phage candidates showed great diversity and divergence and had many pathogenic bacteria as putative hosts, such as Streptococcus suis, Salmonella enterica, and Klebsiella pneumoniae (Fig. 4), suggesting that the abundant bacteriophage composition in the pig virome is of potential value in the exploration for new antibacterial agents.

The present study has revealed the viromic signatures and viral tropisms in the respiratory, alimentary, and circulatory systems of pigs and found that while there was a clustering of viral species in samples of the alimentary and circulatory systems in all farms, the viromic composition of the respiratory tract was much more divergent while sharing some similarities with the viromes of the alimentary and circulatory systems (Fig. 5). This might be ascribed to the ready infectibility of the pharynx with its connections to the alimentary, respiratory, and circulatory systems, through which viruses will go separate routes based on their tropisms to different physiological systems after primary infection.

Notwithstanding the frequent and cross-boundary movement of animals, the pig virome did not show relatedness to geographic locations (Fig. S3). It has been shown that pig transportation plays a critical role in the dissemination of a variety of pathogens (39–42). It is well known that management and biosafety are crucial to maintain health pig farming, but how these factors affect the pig virome remained unclear. Here, we introduced a series of covariates and found that environmental factors, excluding the surroundings, did impact the viromic composition to various degrees (Fig. 6 and 7). In particular, administration, isolated housing, and disinfection all had highly significant impacts on the eukaryotic virome, with immunization, house hygiene, quarantine, and air quality following in importance. The most striking finding was that nonpathogenic resident viruses predominantly circulated in high-scoring farms, while pathogenic nonresident viruses were more often found in low-scoring ones (Fig. 6D to F). Interestingly, some covariates, especially disinfection, showed extremely significant impacts on the prokaryotic virome (Fig. 7), indicating that biosafety measures can exert an influence on the prokaryotic virome via bacteria. The present study is the first to show how high standards of animal farm management with strict biosafety measures can significantly minimize the risk of the introduction of pathogenic viruses into pig farms.

Although the knowledge that pigs are important hosts of zoonotic viruses (10–14) raises major concerns about their roles in public health, the pig virome described here did not identify known zoonotic viruses, consistent with negative findings in previous virome studies (16–18, 43), thereby providing some reassurance that pigs are not necessarily important reservoirs of zoonotic viruses and will rarely transmit zoonotic viruses to humans, as long as their feeding is well managed and disease control measures are well implemented. Currently there are more than 60 viruses known to be pathogenic in pigs (15), but new viruses or mutated ones continue to emerge. As described above, 17.6% of mammalian viral contigs were identified as new viruses but have previously received little attention. These viruses may have significant implications for the prediction of future emerging diseases, especially PCV4, which will require further investigation to assess its pathogenicity, which was also recently reported in piglets with respiratory disease and diarrhea in several Chinese farms (24, 44).

In veterinary clinics, prevention and control of infectious diseases is a main task that requires timely surveillance of the mutated, new, exotic, and zoonotic viruses. Since traditional methods depend largely on the specific detection of known pathogens, development of countermeasures is generally outpaced by the spread of newly emerging viruses. Our results showed that viromic profiling in disease surveillance could successfully identify risk viruses, therefore resulting in a new concept of “precision surveillance” that enables high-throughput detection and identification of all known and even unknown pathogenic viruses at large scale in a single test. Compared to the limited capacity of traditional methods that depend on detection of specified pathogens, this viral metagenomic-based surveillance could be an innovative supplement and application in the clinical veterinary setting, which also enables identification of mixed infections and differentiation of virus variants and field viruses from vaccine strains. To facilitate the application of this new concept in animal viral disease prevention and control, the viromic pipeline (Fig. S1) was proposed, in which we incorporated filtration through a 0.22-μm-pore-size filter in sample preparation prior to sequence-independent amplification (SIA). Filters with pore sizes of 0.22 or 0.45 μm are widely used in viromic studies to remove host cell debris and bacteria. Here, we focused on the majority of pathogenic viruses that are almost all smaller than 0.22 μm, which allowed us to generate high proportions (∼20%) (Data Set S1) of VLSs in total reads. However, if the whole virome or large viruses are main targets, filtration through a 0.45-μm-pore-size membrane or even larger is highly recommended (45). Currently, viromic techniques include RNA-specific methods, i.e., SIA and meta-transcriptomics (MTT) (46, 47), and DNA-specific methods, i.e., multiple displacement amplification (MDA) and metagenomics (MTG) (48, 49). Of them, MTT, MDA, and MTG are highly nucleic acid (NA) specific. If a study focuses on both DNA and RNA viromes, MTT with either MDA or MTG is necessary, which undoubtedly increases the cost in clinical use. Notwithstanding, SIA is usually used for the RNA virome (46), since reverse transcriptase used in the synthesis of cDNA has the activity of DNA-directed DNA polymerase, which can synthesize the counterpart of single-stranded DNA (ssDNA) (50), and hence, SIA is also effective in detection of DNA viruses. It is why DNA virus reads in the present study were not dwarfed, accounting for 96% of the total virome. Published SIA-based viromes also detected abundant DNA viruses (51, 52). However, since bias is prone to be introduced by SIA during the random amplification of NA, which could result in fragmentation of the viral genome, effective assembly of complete viral genomes could be hampered. In this regard, the unbiased viromic methods, especially MTT and MTG, would be valuable alternatives. Given that the viromic approach has been increasingly used to tackle human diseases (53), but rarely in veterinary medicine, this strategy should innovate currently used surveillance methods of animal infectious diseases, particularly by making precision surveillance available on a large scale or even during a nationwide surveillance campaign.

## MATERIALS AND METHODS

### Sample and data collection.

The procedures for sample collection of pigs in this study were viewed and approved by the Administrative Committee on Animal Welfare of the Institute of Military Veterinary Medicine, Academy of Military Medical Science, China. All pigs were maintained and handled according to the *Principles and Guidelines for Laboratory Animal Medicine* of the Ministry of Science and Technology, China (54). To obtain a representative viromic profile of the pig population, the sample collection was focused on commercial pig farms in 25 major pig-producing regions across China (Fig. 1). In each region, 1 to 5 pig farms were selected for sampling based on the following conditions: (i) had more than 3 years of pig-farming history; (ii) had at least 100 producing sows or annual slaughters of more than 1,000 pigs; (iii) had at least 1 year free of disease outbreak, with all animals in apparent good health during the previous 3 months; and (iv) all pigs were cross-breeds or tertiary breeds produced from foreign breeds (Landrace, Durac, and Yorkshire). In each farm, pharyngeal and anal swabs and 2 ml sera were collected from about 40 35- to 50-day-old weaned piglets. Samples were immediately transported to the laboratory in dry ice and stored at −80°C pending further use. All sampled farms were examined by an investigator for pig health status. The owners or veterinarians of participating farms were asked to sign a contract which guaranteed anonymity of all collected data. During sample collection, a two-part questionnaire (see Table S1 in the supplemental material) was completed by interviewing the farm owner or veterinarian. The first part included questions about general farm characteristics, and 8 scoring systems were adapted from Biocheck.UGent (http://www.biocheck.ugent.be). The second part of the questionnaire included immunization procedures and vaccination of sampled piglets.

### Sample preprocessing and Illumina sequencing.

All samples were subjected to our VIRES procedure (Fig. S1). Equal volumes of pharyngeal and anal swab solutions and sera from each farm were respectively pooled to provide a final volume of 2 ml for each sample type. Since almost all pathogenic mammalian viruses are small in size, the centrifuged supernatants were filtered through 0.22-μm-pore-size membranes, digested with DNase I and RNase A (TaKaRa, Dalian, China), and immediately subjected to extraction of total NA using an RNeasy minikit (Qiagen) without DNA enzymatic digestion. Total NA was subjected to SIA using three barcoded random primers to differentiate sample type. The PCR products of each sample type were purified, and then equal amounts were pooled to provide samples of 2 μg NA. One microgram of pooled qualified DNA from each farm was subjected to Illumina paired-end (125-bp) sequencing with an insert size of 180 bp in a HiSeq 4000 sequencer. The resulting raw reads were quality checked using FastQC v0.11.7 and trimmed using Trimmomatic v0.38 (55), and the results were used for bioinformatics analyses as clean data.

### Virus annotation.

The bioinformatics pipeline of the virome, which was composed of four parts, namely, two steps of quality control, *de novo* assembly, and viral annotation, is illustrated in Fig. S1B. Host genomes were removed from clean data by mapping against Sscrofa11.1 (GenBank accession no. AEMK02000000) using Bowtie 2 v2.4.1 with the sensitive mode (56). The remaining sequence pairs from each farm were subjected to overlapping using FLASH v1.2.11 and barcode removal. The nonbarcode total reads were mixed and assembled *de novo* using MEGAHIT v1.1.3 (57), while unassembled reads were subjected to another round of *de novo* assembly. The final contigs and unused reads were first subjected to reference-based annotation using BLASTn v2.7.1 for searching viral sequences (including noncoding ones) highly similar to known viruses and DIAMOND v0.9.25 for searching viral sequences with limited similarities to known viruses with an E value under 1e−5 against a customized viral nucleotide reference database of GenBank (Taxonomy ID 10239, version 29/09/2019) and the UniProt (release 2019_07) virus taxonomic database, respectively. To uncover remote sequences related to viruses, total contigs were subjected to putative protein translation using Prodigal v2.60 with –g 1 and –p meta (58). The generated amino acid (aa) sequences (≥50 aa) were scanned against three HMM profiles: the eukaryotic virus-exclusive vFam (59), the prokaryotic virus orthologous groups (pVOG) (60), and the virus branch of eggNOG v5.0 (61), using hmmscan of HMMER v3.2.1 with an E value of 1e−5. The BLASTn-, DIAMOND-, and HMMER-generated sequences were retained as putative VLSs if they met the following criteria: (i) were shared by the three methods with the same or close taxonomic annotation, and/or (ii) had no significant hits to nonvirus nt/nr databases by BLASTn/x searches (E value ≥ 1e−20; identity ≤ 50%).

### Quality control.

The possibility of contamination introduced by intersampling, commercial kits, and the environment was assessed by two methods. First, during sample preparation and sequencing in this study, we also conducted viral metagenomic analyses of bats, ticks, and rodents, using the same protocol with the same commercial kits and reagents. Their Illumina sequencing-generated clean data (2 bat data sets [FM1701 and XB1703; accession no. SRR9644024 and SRX6405658] [62], 1 tick data set [TK1612; accession no. SRR7985010] [63], and 1 rodent data set [XJ1607; accession no. SRP126625] [64]) were used as control data and annotated by the same pipeline (we did not use any blank negative controls, since sequencing library construction of them often failed [65]), and their VLSs were used as subjects in a BLASTn search of merged reads in this study. Moreover, reads of control data were also searched against VLSs in the PVD. BLASTn hits with ≥98% identity and ≥80% coverage were considered false positives. Besides, possible FPSs of viruses reported from nucleic acid extraction spin columns and laboratory components were retrieved from GenBank (20, 22). These FPSs were matched to the putative VLSs, with BLASTn hits showing ≥98% identity and ≥80% coverage being considered contaminants. All FPSs identified were removed from the putative VLSs, and the remaining VLSs were further condensed using CD-HIT v4.5.4 at an identity of 0.95 and alignment coverage for the shorter sequence of 0.95, which generated the final data set, named Pigs_VIRES.

### Genus-level taxonomy assignment.

The Pigs_VIRES virome was divided into eukaryotic and prokaryotic sub-data sets according to their reference- and HMM-based annotations. Merged with prokaryotic viral RefSeq database release 94 sequences, the prokaryotic virome was subjected to an all-versus-all DIAMOND search and then to protein clustering using MCL and virus clustering using ClusterONE v2 run under the vConTACT2 pipeline, a prokaryotic virus genome clustering tool, with default settings (66). The generated network was visualized with Cytoscape v3.6.1 using yFiles Organic Layout. Taxonomic assignment of the eukaryotic virome was carried out using MCL, and the obtained eukaryotic viromic sequences were merged with the eukaryotic virus sequences in RefSeq database release 97 and then subjected to protein clustering using MCL with an inflation factor (IF) of 7.0, at which clustering of reference sequences showed the highest coincidence with ICTV genus classification when assessed using *Coronaviridae*, *Picornaviridae*, *Reoviridae*, *Parvoviridae*, and *Adenoviridae*.

### Assessment of viromic structure.

To assess the viromic structure of Pigs_VIRES, 19 previously reported high-throughput viromic sequence data sets representing pigs (*n* = 4), takins (*n* = 1), cattle (*n* = 2), goats (*n* = 1), ducks (*n* = 1), broiler chickens (*n* = 7), birds (*n* = 1), and humans (*n* = 2) from Canada, China, India, Hungary, Sweden, the United States, and Venezuela were downloaded from the NCBI Sequence Read Archive (SRA) database. Detailed information about these, with references, is summarized in Data Set S2. They were subjected to viromic analyses using the above-described pipeline. Their results were merged into four data sets for pigs, bovines (including cattle, takins, and goats), avians (including ducks, broilers, and birds), and humans according to their hosts. These data sets were merged with Pigs_VIRES and subjected to vConTACT2 analysis to cluster proteins.

### Host prediction of bacteriophages.

The prokaryotic virome was subjected to prediction of likely bacterial hosts. Sequences were first mapped against the microbe-versus-phage (MVP) phage genomic clusters using BLASTn, and matches were retained if the identity was at least 95% and coverage was ≥80% (25). The host was then determined from its matching viral cluster (25). These sequences were also subjected to a BLASTn search for CRISPR spacer matches under the blastn-short model with an E value of <1e−5. The CRISPR spacer subjects were composed of two parts, one from the spacer collection in the CRISPRdb database with 221,397 spacers as of the 18 June 2019 release (67), and the other with 720,391 spacers predicted from archaeal and bacterial genomic sequences using CRISPRFinder and PILER-CR (68).

### Ecological and statistical analyses.

Ecological and statistical analyses were conducted in the R environment (v3.5.1; http://www.r-project.org/). To determine the interfarm beta diversity of eukaryotic and prokaryotic viromes, reads were counted by mapping against Pigs_VIRES using a BLASTn search with an identity of ≥99% and coverage of ≥90%. In order to minimize bias in the ratios of viral NA composition introduced by pooling strategy and PCR amplification, binary statistics of annotation were Hellinger transformed and subjected to PCA analysis. Agglomerative hierarchical clustering based on the calculated Jaccard distances was performed under the ward.D strategy. Hellinger-transformed annotation was detrended with distance-based geodetic coordinates. The distance matrix of residuals was used for Mantel correlogram analysis with 10,000 permutations. The principal coordinates of neighborhood matrix (PCNM) analysis of spatial distances was also computed and analyzed with detrended annotation using redundancy analysis. To determine significant covariates, the effect size and significance of each covariate were determined using the envfit function in vegan, comparing the difference in the centroids of each group relative to the total variation (69). In total, eight covariates with known associations to husbandry maintenance were included in the envfit analysis.

### Virus detection.

DNA of involved samples was extracted as described above. Nested or seminested PCR assays were carried out to determine the prevalence of retrovirus and circovirus. Primers were designed based on the alignments of contigs with their reference sequences. PCR was performed with 2× PCR MasterMix (Tiangen) under the following cycling conditions: denaturation at 94°C for 30 s, annealing at 57°C for 30 s, and extension at 72°C for 40 s.

### Sequence comparison.

To identify whether the sequences in Pigs_VIRES contained vaccine viruses, the complete genomes of the most widely used attenuated vaccine strains were used as subjects of a BLASTn search: PEDV AJ1102 (Genbank accession no. JX188454/MK584552), PEDV LW/L (MK392335), transmissible gastroenteritis virus (TGEV) WH-1 (HQ462571), porcine reproductive and respiratory syndrome virus (PRRSV) CH-1a (AY032626), PRRSV CH-1R (EU807840), PRRSV GD (EU825724), PRRSV HUN4 (EF635006), PRRSV JXA1-P80 (FJ548853), CSFV HCLV (Z46258), and PRV Bartha (JF797217). Pigs_VIRES sequences were considered to have originated from vaccine strains if they showed 99% nucleotide identity and 90% coverage with targeted vaccine strains. To investigate the phylogenetic relationships of selected viruses, the longest contigs or contigs at the same genomic locations from different farms were aligned with representative sequences using MAFFT version 7.0 (70), and phylogenetic analyses were conducted by maximum-likelihood analysis, with the best-fit substitution model and 100 bootstrap replicates in PhyML version 3.3 (71). Phylogenetic trees were visualized with FigTree v1.4.3. Sequence identities were calculated by MegAlign v3.3.8 (DNASTAR).

### Data availability.

All sequence reads generated in this study are available in the NCBI SRA database under BioProject accession number PRJNA512250. Representatives of assembled contigs with genetic novelty and of complete genomes have been deposited in GenBank under accession numbers MK377447 to MK379577, MK948405 to MK948415, and MT135230 to MT135500. The complete genome and amplicons of PCV4 identified here have been deposited in GenBank under accession numbers MK948416 to MK948425. The Pigs_Virome in FASTA format containing Pigs_VIRES and Pigs_GenBank, the phylogenetic trees in newick format, and key codes to produce results and figures are available from Figshare at https://doi.org/10.6084/m9.figshare.9211265.v1.
