# Supplementary figures and images for: Viral Metagenome-Based Precision Surveillance of Pig Population at Large Scale Reveals Viromic Signatures of Sample Types and Influence of Farming Management on Pig Virome
Source: mSystems. 2021 Jun 8;6(3):e00420-21. doi: 10.1128/mSystems.00420-21 (PMC8269232; doi:10.1128/mSystems.00420-21)

A

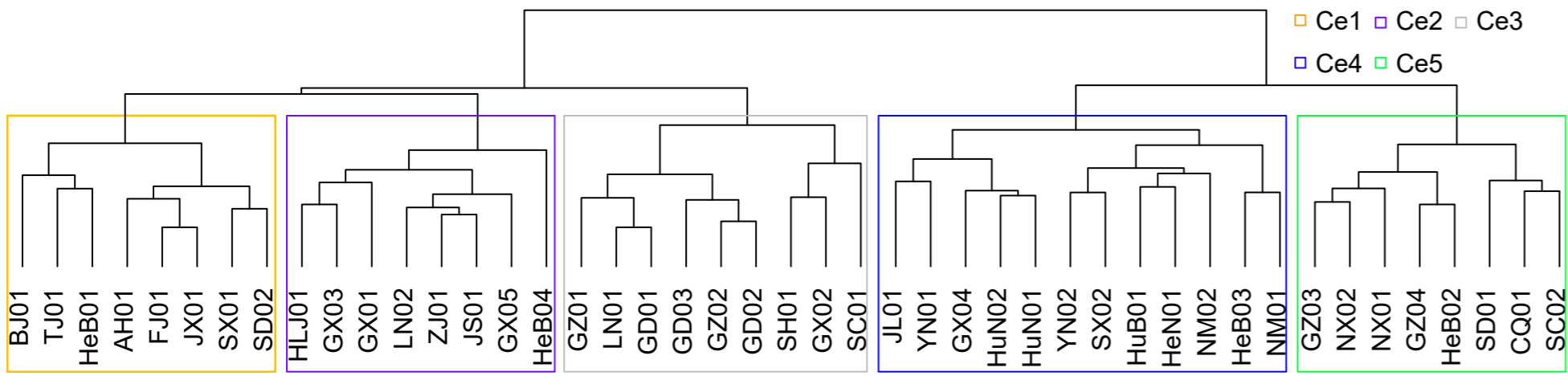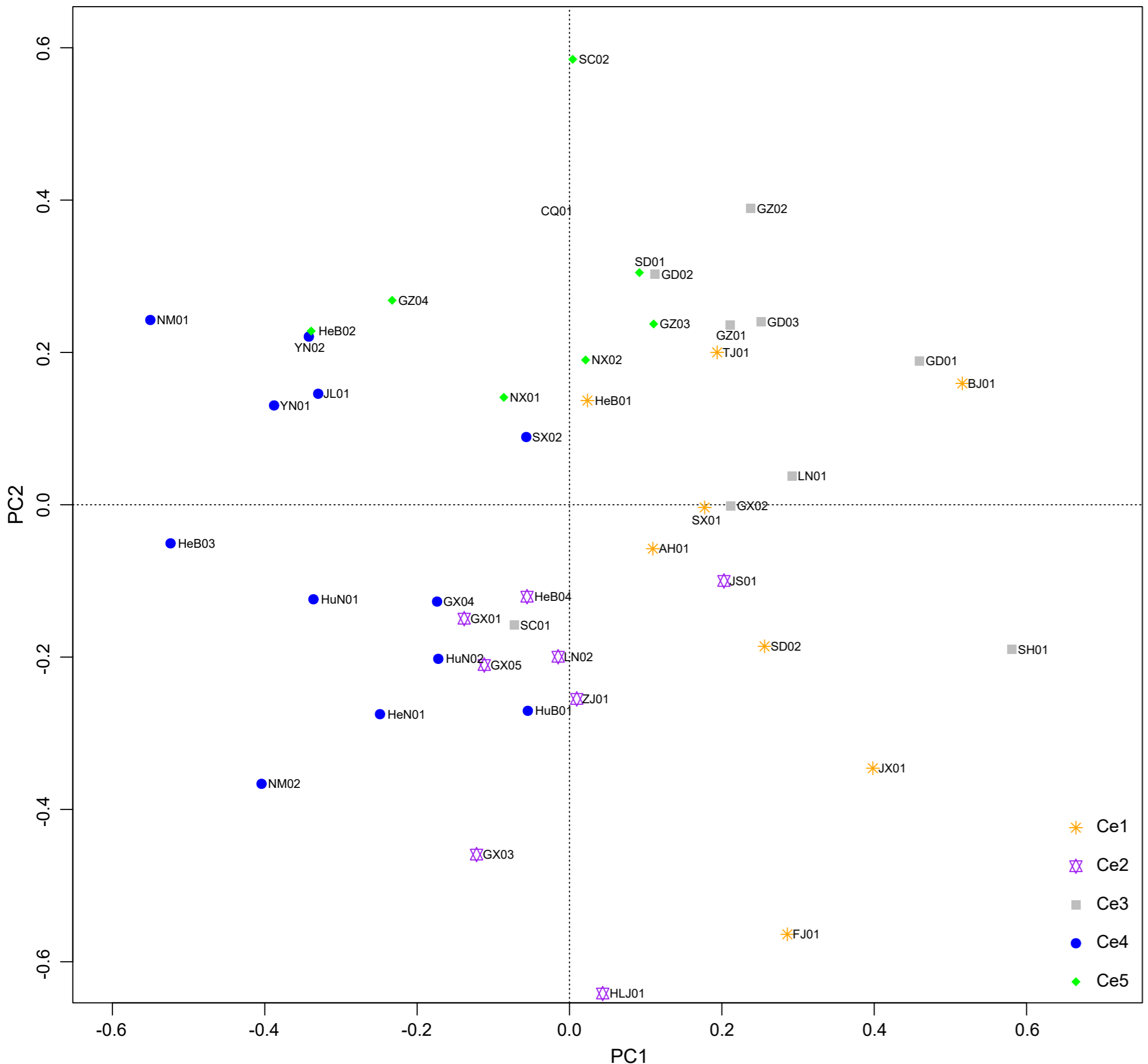

B

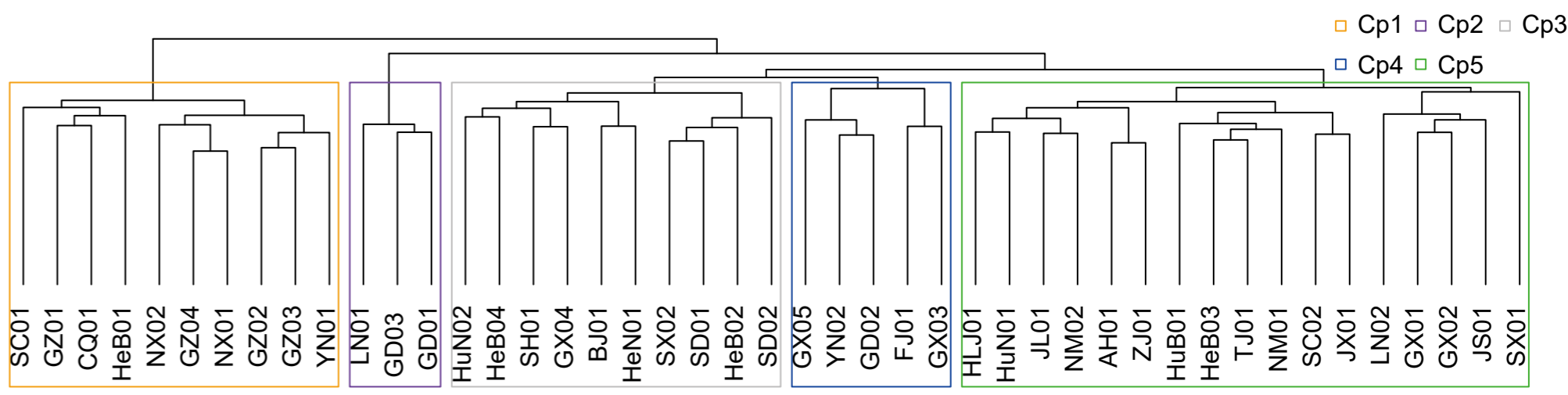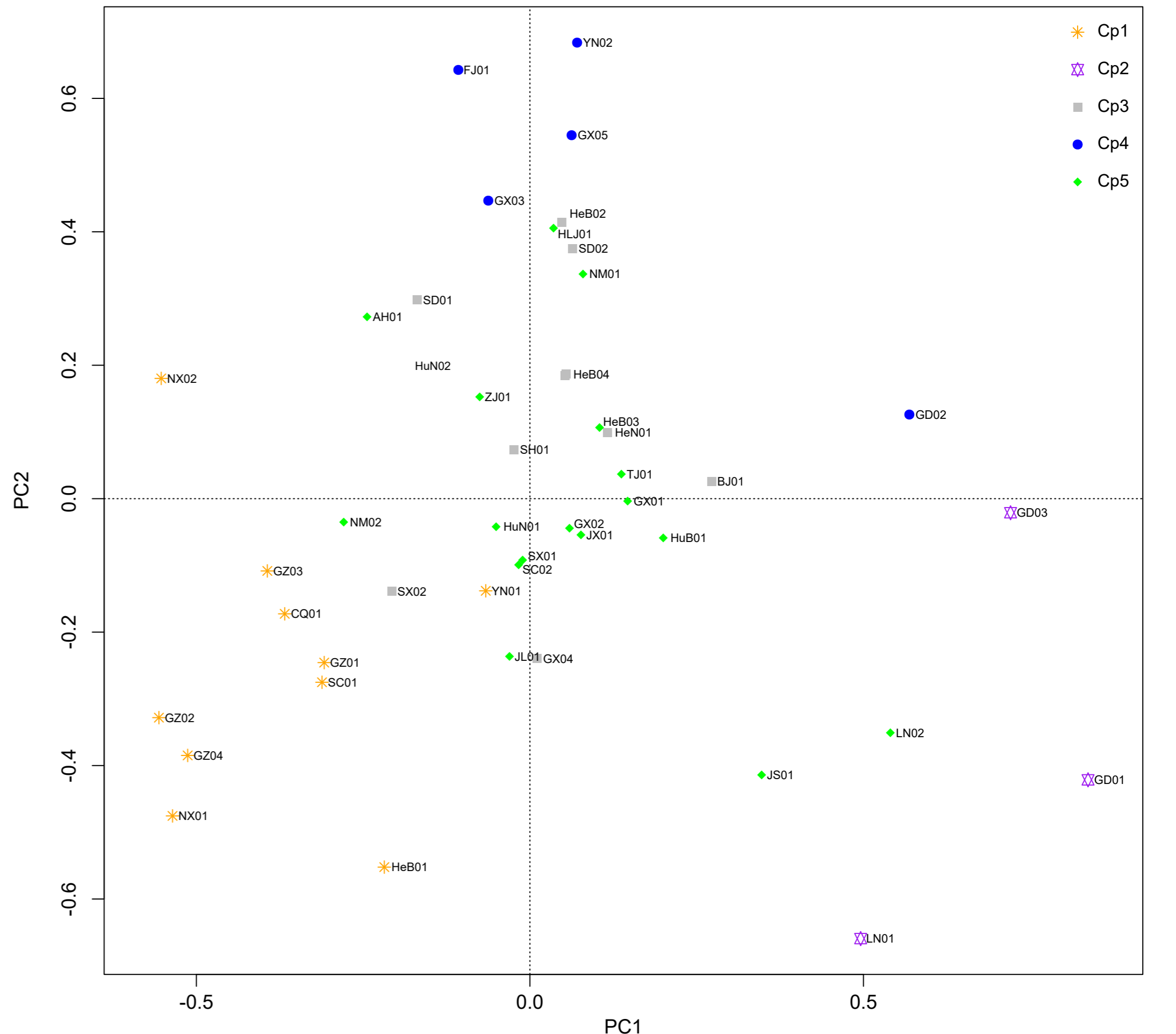

Supplement: FIG S3 [file msystems.00420-21-sf003.pdf]
