# Supplementary material for: Viral Metagenome-Based Precision Surveillance of Pig Population at Large Scale Reveals Viromic Signatures of Sample Types and Influence of Farming Management on Pig Virome
Source: mSystems. 2021 Jun 8;6(3):e00420-21. doi: 10.1128/mSystems.00420-21 (PMC8269232; doi:10.1128/mSystems.00420-21)
Supplement: TABLE S1 [file msystems.00420-21-st001.pdf]

Table S1. Questionnaire of management and biosafety measures

Category	Items	Questions	Ranks
Management	Administration	Does the farm establish a sound management system? Does the farm have reasonable staffing, especially veterinarian? Is staff well-trained?	◯Excellent<br>◯Good<br>◯Inferior
	Isolated housing	Are pigs at breeding, nursing and fattening reared in separate houses?	◯Excellent<br>◯Good<br>◯Inferior
	House hygiene	Are all pig houses cleaned regularly?<br>Is there control of flying pests (mosquitos and flies, etc) and rats?<br>Do all pig houses have equipment for excreta disposal?	◯Excellent<br>◯Good<br>◯Inferior
	Air quality	Is there any equipment for temperature and humidity control? Does the air in all farms circulate well?	◯Excellent<br>◯Good<br>◯Inferior
	Surroundings	Is the farm located in an area separate from any human residence, other farm animals and traffic lines within 5 km?	◯Excellent<br>◯Good<br>◯Inferior
Biosafety	Immunization measures	Are there routine immunization measures in place?<br>Are the procedures strictly conducted?	◯Excellent<br>◯Good<br>◯Inferior
	Disinfection	Is there any equipment for disinfection of staff, visitors and vehicle at the entrance of the farm?<br>Are all pig houses regularly disinfected and emptied for days after a feeding cycle?	◯Excellent<br>◯Good<br>◯Inferior
	Quarantine	Is there any protocol for, and separated room, for quarantine when breeding pigs or piglets are introduced?	◯Excellent<br>◯Good<br>◯Inferior
